# Metal-dependent programmed cell death-related lncRNA prognostic signatures and natural drug sensitivity prediction for gastric cancer

**DOI:** 10.3389/fphar.2022.1039499

**Published:** 2022-10-21

**Authors:** Xuesong Song, Lin Hou, Yuanyuan Zhao, Qingtian Guan, Zhiwen Li

**Affiliations:** ^1^ Department of Anesthesiology, First Hospital of Jilin University, Changchun, China; ^2^ First Hospital of Jilin University, Changchun, China

**Keywords:** gastric cancer, prognostic prediction, natural drug sensitivity, metal-dependent programmed cell death, immune sensitivity

## Abstract

**Background:** Gastric cancer is one of the most important malignancies with poor prognosis. Ferroptosis and cuproptosis are newly discovered metal-dependent types of programmed cell death, which may directly affect the outcome of gastric cancer. Long noncoding RNAs (lncRNAs) can affect the prognosis of cancer with stable structures, which could be potential prognostic prediction factors for gastric cancer.

**Methods:** Differentially expressed metal-dependent programmed cell death (PCD)-related lncRNAs were identified with DESeq2 and *Pearson*’s correlation analysis. Through GO and KEGG analyses and GSEA , we identified the potential effects of metal-dependent PCD-related lncRNAs on prognosis. Using Cox regression analysis with the LASSO method, we constructed a 12-lncRNA prognostic signature model. Also, we evaluated the prognostic efficiency with Kaplan–Meier (K-M) survival curve, receiver operating characteristic (ROC) curve, and decision curve analysis (DCA) methods. The sensitivities for antitumor drugs were then predicted with the pRRophetic method. Also, we discuss Chinese patent medicines and plant extracts that could induce metal-dependent programmed cell death.

**Results:** We constructed a metal-dependent PCD-related lncRNA-gene co-expression network. Also, a metal-dependent PCD-related gastric cancer prognostic signature model including 12 lncRNAs was constructed. The K-M survival curve revealed a poor prognosis in the high-risk group. ROC curve analysis shows that the AUC of our model is 0.766, which is better than that of other published models. Moreover, the half-maximum inhibitory concentration (IC50) for dasatinib, lapatinib, sunitinib, cytarabine, saracatinib, and vinorelbine was much lower among the high-risk group.

**Conclusion:** Our 12 metal-dependent PCD-related lncRNA prognostic signature model may improve the OS prediction for gastric cancer. The antitumor drug sensitivity analysis results may also be helpful for individualized chemotherapy regimen design.

## Introduction

Gastric cancer is one of the most important malignancies worldwide, resulting in unhealthy dietary habits, increasing social burden, and *helicobacter pylori* infection. Further, 1,089,103 new cases and 768,793 death cases occurred in 2020 (Sung et al., 2021). Although surgical methods and adjuvant therapeutic technologies have improved rapidly, the outcome of gastric cancer patients has not improved. Especially when progressed to an advanced status, the 5-year overall survival (OS) rates remained lower than 20 percent (Ferlay et al., 2019; Tan, 2019). At present, prognostic evaluation is referred to the tumor node metastasis (TNM) classification standard. But the drug response and individual variation would also affect the outcome of gastric cancer, due to the tumor mutation burden (TMB) (Chen et al., 2022; Lee et al., 2022) and drug-resistant status (Wang et al., 2022a; Blange et al., 2022; Zhong et al., 2022). Therefore, it is urgently needed to identify novel powerful prognostic markers and to predict the drug sensitivity status for gastric cancer.

Ferroptosis and cuproptosis are newly discovered metal-dependent types of programmed cell death (PCD) distinct from apoptosis and autophagy (Dixon et al., 2012; Tang et al., 2019; Wang et al., 2022b). Through the accumulation of reactive oxygen species (ROC), ferroptosis controls cell death by the dysregulation of glutathione peroxidase activities (Li et al., 2020). Meanwhile, cuproptosis is a copper-triggered modality of mitochondrial cell death, which may be also mediated by lipoylated TCA cycle proteins (Cobine and Brady, 2022; Li et al., 2022; Tang et al., 2022). Recent studies have shown that the aberrant expression of ferroptosis- and cuproptosis-related genes are critical risk factors that directly affect the prognosis of cancer patients (Xie et al., 2016; Roemhild et al., 2021). Moreover, long noncoding RNAs (lncRNAs) are special endogenic functional molecules, which regulate the metabolic progress of gastric cancer cells (Ransohoff et al., 2018). The aberrant expression of lncRNAs regulates the functions of ferroptosis- and cuproptosis-related genes (Mao et al., 2018; Wang et al., 2019; Wu et al., 2020; Zhang et al., 2022a), which are potential prognostic biomarker pools (Statello et al., 2021).

In our study, we collected the RNA-seq, clinicopathological, survival, and simple nucleotide variation data of stomach adenocarcinoma (STAD) from The Cancer Genome Atlas (TCGA) database. Also, we screened the differentially expressed (DE) genes and lncRNAs. Then, we constructed the metal-dependent PCD-related lncRNA-gene co-expression network. GO and KEGG analyses and GSEA showed that these lncRNAs affect the patients’ prognosis status. A 12-lncRNA signature model for gastric cancer prognostic evaluation was built based on metal-dependent PCD-related lncRNAs. Receiver operating characteristic (ROC) curve analysis shows that the AUC value is 0.766. Also, drug sensitivity analysis showed that the half-maximum inhibitory concentration (IC50) for dasatinib, lapatinib, sunitinib, cytarabine, saracatinib, and vinorelbine was much lower among the high-risk group. Therefore, the prognostic evaluation model constructed in our study may improve the prognostic prediction for gastric cancer patients.

## Materials and methods

### Data collection

We collected the open RNA-seq data, clinicopathological data, OS information, and simple nucleotide variation data for stomach adenocarcinoma (STAD) and para-carcinoma tissues from The Cancer Genome Atlas (TCGA) database (https://cancergenome.nih.gov/) (Hutter and Zenklusen, 2018). Totally, transcriptome data for 434 tissues (387 STAD tissues and 47 normal tissues from 387 patient samples) were downloaded. Then, in order to annotate the RNA-seq data, we also collected the TCGA annotation information version 22 from the GENCODE database (https://www.gencodegenes.org/) (Frankish et al., 2019). The ferroptosis-related gene list was downloaded from the FerrDb database (http://www.zhounan.org/ferrdb/), and the cuproptosis-related gene list was collected among all published reports (Zhou and Bao, 2020) (detailed in Supplementary Table S1). In addition, the gene sets used for gene set enrichment analysis (GSEA) were collected from MSigDB (http://www.gsea-msigdb.org/gsea/index.jsp) (Subramanian et al., 2005).

### Identification of metal-dependent programmed cell death-related differentially expressed lncRNAs

“DESeq2” R package was used to identify the DE lncRNAs and DE genes under R version 4.0.4. Moreover, lncRNAs or genes with the threshold values |log2(FoldChange)| > 1 and *p*-value < 0.05 were defined as DE. Then, we used *Pearson*’s correlation analysis to identify the co-expression relationships, and lncRNAs with threshold values |cor| > 0.7 and *p*-value < 0.05 were defined as co-expressed.

### Construction for the metal-dependent programmed cell death-related lncRNA-gene co-expression networks

Utilizing Cytoscape version 3.8.3, we visualized the metal-dependent programmed cell death lncRNA-gene co-expression network. Also, the edge widths represent *Pearson*’s correlation coefficient. The node sizes represent the betweenness centrality for each molecule.

### Functional analysis of the metal-dependent programmed cell death-related lncRNA-gene co-expression networks

The “clusterProfiler” R package was used to identify the functions of the co-expression networks involved under R version 4.0.4. We performed Gene Ontology (GO) annotation to identify the functions for each gene and Kyoto Encyclopedia of Genes and Genomes (KEGG) pathway enrichment analysis to identify the significantly involved metabolism pathways, with default parameters. Then, we performed gene set enrichment analysis (GSEA) with the gene set “h.all.v7.5.1.entrez.gmt” downloaded from MSigDB.

### Construction of a metal-dependent programmed cell death-related lncRNA prognostic model

The “survival” and “glmnet” R packages were used to perform Cox regression with the least absolute shrinkage and selection operator (LASSO) algorithm and multivariate Cox regression analysis under R version 4.0.4. Then, utilizing the prognostic model, risk scores were calculated with the formula $Risk\;Scores=\overset{n}{\underset{1}{\sum }}{Cor}_{lncRNAi}\times {\mathrm{Exp}}_{lncRNAi}$ (where Cor_lncRNAi_ represents the correlation coefficient of interfering lncRNAi, Exp_lncRNAi_ represents the expression level for lncRNAi, and n represents the number of lncRNA signatures). Then, the patients were divided into high-risk group (over the median risk score) and low-risk group (no more than the median risk score).

### Nomogram and calibration analysis

The “rms” R package was used to construct the nomogram in order to predict the 1-, 3-, and 5-year OS rates of gastric cancer patients under R version 4.0.4. “rmda” R package was then used to build the calibration curve and evaluate the consistency between the OS rates predicted by the nomogram and the actually observed OS rates (Zhang and Kattan, 2017).

### Survival and reactive oxygen species analysis

The “survival” and “survminer” R packages were used to perform the Kaplan–Meier (K-M) survival curve analysis on the OS rates under R version 4.0.4. Also, the “survivalROC” R package was also used for ROC analysis.

### Tumor mutation burden analysis

Tumor mutation burden (TMB) is the total exonic mutation count per megabase of tumor DNA. Using homemade Perl script, we calculated the tumor mutation burden (TMB) scores for each STAD sample. The difference in TMB scores between the high-risk and low-risk group was calculated with R version 4.0.4.

### Immune functions and immunotherapy prediction

The “limma,” “GSVA,” “GSEABase,” and “ggpubr” R packages were used to evaluate the immune escape functions and immunotherapy difference between the high-risk and low-risk groups, which were divided according to the predicted results of PCD-related lncRNA under R version 4.0.4.

### Drug sensitivity prediction

The “pRRophetic” R package (Geeleher et al., 2014) was used to predict the clinical drug sensitivities for antitumor drugs under R version 4.0.4. The correlation between risk scores and half-maximum inhibitory concentration (IC50) was evaluated. Also, the difference in drug response for high-risk and low-risk groups was exhibited with the bar plot.

## Results

### Identification of metal-dependent programmed cell death-related differentially expressed lncRNAs

Following the workflow shown in Supplementary Figure S1, we totally identified 4466 DE genes (2125 upregulated and 2341 downregulated), as well as 3391 DE lncRNAs (2394 upregulated and 997 downregulated), detailed in Supplementary Table S2. Among these genes, 84 DE metal-dependent PCD-related genes were identified (23 upregulated and 61 downregulated, detailed in Supplementary Table S3). Through *Pearson’*s correlation analysis (|cor| ≥ 0.7 and *p*-value < 0.05), we identified 271 metal-dependent PCD-related DE lncRNAs among gastric cancer tissues (Supplementary Table S4).

**FIGURE 1:**
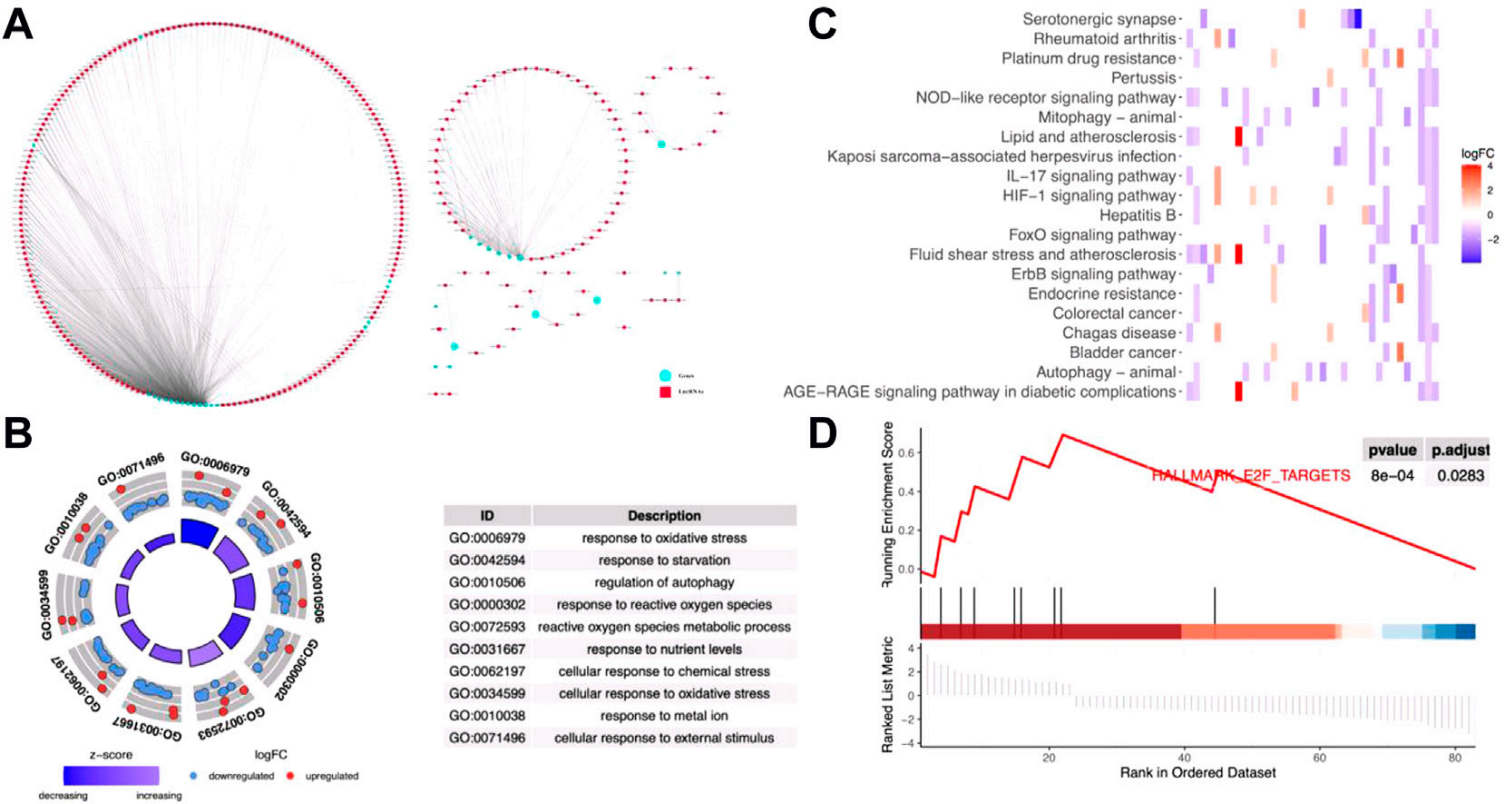
Functions of metal-dependent programmed cell death-related genes. **(A)** lncRNAs-mRNAs co-expression network. *Blue circles* represent differentially expressed ferroptosis- and cuproptosis-related genes among gastric cancer tissues. *Red squares* represent co-expressed lncRNAs with relative coefficient over 0.7. **(B–D)** Gene Ontology (GO) annotation **(B)** and Kyoto Encyclopedia of Genes and Genomes (KEGG) enrichment analyses **(C)** and gene set enrichment analysis (GSEA) **(D)** were performed among genes involved in the co-expression network.

In order to identify the potential effects of metal-dependent PCD-related lncRNAs on gastric cancer outcomes, we built a lncRNAs-genes co-expression network (Figure 1A, detailed in Supplementary Table S4). GO annotation shows that the genes involved in this network played important roles in “GO:0010038 response to metal ion,” “GO:0006979 response to oxidative stress,” and “GO:0072593 reactive oxygen species metabolic process” (Figure 1B, detailed in Supplementary Table S5). Also, the “HIF-1 signaling pathway,” “FoxO signaling pathway,” and “ErbB signaling pathway” were significantly enriched with the KEGG pathway enrichment analysis (Figure 1C, detailed in Supplementary Table S6). Moreover, GSEA showed “HALLMARK E2F targets” which was also significantly identified (Figure 1D). All these results mentioned earlier indicated that the metal-dependent PCD-related lncRNAs would regulate the prognosis of gastric cancer by controlling the activities of oncogenic pathways, which could be potential prognostic prediction features.

### Construction of metal-dependent programmed cell death-related prognostic prediction lncRNA signatures

**FIGURE 2:**
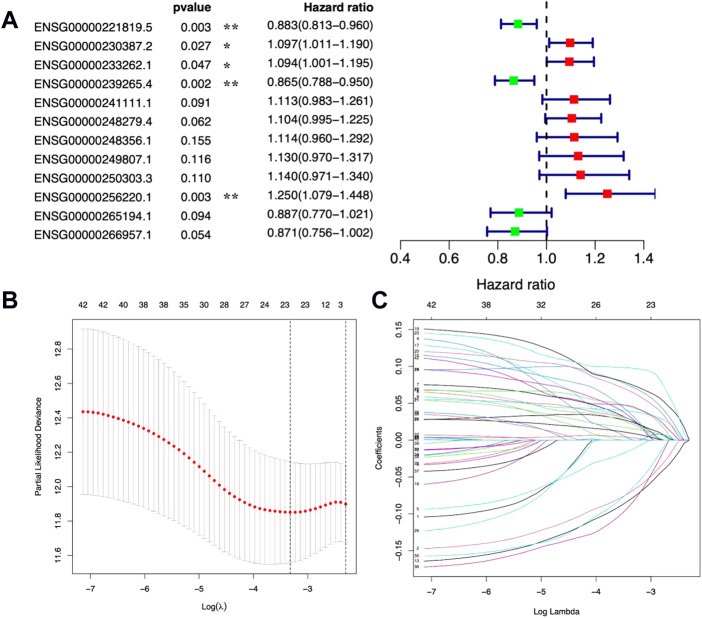
Prognostic values of 12 metal-dependent programmed cell death-related lncRNAs. * *p*-value < 0.05 and ** *p*-value < 0.01. **(A)** Forest plot for prognostic values. **(B,C)** Cv-fit **(B)** and lambda **(C)** values for the prognostic prediction model.

Then, based on the expression levels of the 271 DE metal-dependent PCD-related lncRNAs and OS information, a gastric cancer prognostic prediction model was identified by Cox regression analysis with the LASSO method (Figure 2 and Supplementary Figure S2 for univariated Cox regression analysis). In this model, 12 metal-dependent PCD-related lncRNA signatures were involved in gastric cancer prognostic evaluation (Table 1), including seven prognostic risk factors (ENSG00000230387.2, ENSG00000233262.1, ENSG00000241111.1, ENSG00000248279.4, ENSG00000248356.1, ENSG00000249807.1, and ENSG00000250303.3) and four prognostic protective factors (ENSG00000221819.5, ENSG00000239265.4, ENSG00000265194.1, and ENSG00000266957.1).

**TABLE 1:** Cox regression analysis with the LASSO algorithm for gastric cancer prognostic model.

LncRNA ID	Coefficient	HR	HR.95L	HR.95H	*p*-value
ENSG00000221819.5	−0.123864	0.88349997	0.8130863	0.9600115	0.00346648
ENSG00000230387.2	0.09235646	1.0967557	1.01059988	1.19025649	0.02692752
ENSG00000233262.1	0.08955983	1.09369277	1.00113665	1.1948058	0.04712845
ENSG00000239265.4	−0.1447247	0.8652605	0.787975	0.95012625	0.00243212
ENSG00000241111.1	0.10724518	1.11320716	0.98286698	1.26083204	0.0914179
ENSG00000248279.4	0.09879774	1.10384301	0.99501448	1.22457453	0.0620991
ENSG00000248356.1	0.10779476	1.11381912	0.95987756	1.29244925	0.15549629
ENSG00000249807.1	0.1224871	1.13030454	0.97013481	1.31691838	0.11616748
ENSG00000250303.3	0.13135418	1.14037161	0.97077099	1.33960266	0.10984779
ENSG00000256220.1	0.22291816	1.24971829	1.078681	1.44787553	0.0029919
ENSG00000265194.1	−0.1202328	0.88671397	0.77022369	1.0208225	0.09429351
ENSG00000266957.1	−0.1385471	0.87062223	0.75619881	1.00235951	0.05395695

LASSO, least absolute shrinkage and selection operator; HR, hazard ratio; HR.95L, hazard ratio with lower 95% confidence index; and HR.95H, hazard ratio with high 95% confidence index.

### Prognostic evaluation of the 12 metal-dependent programmed cell death-related lncRNA signatures

**FIGURE 3:**
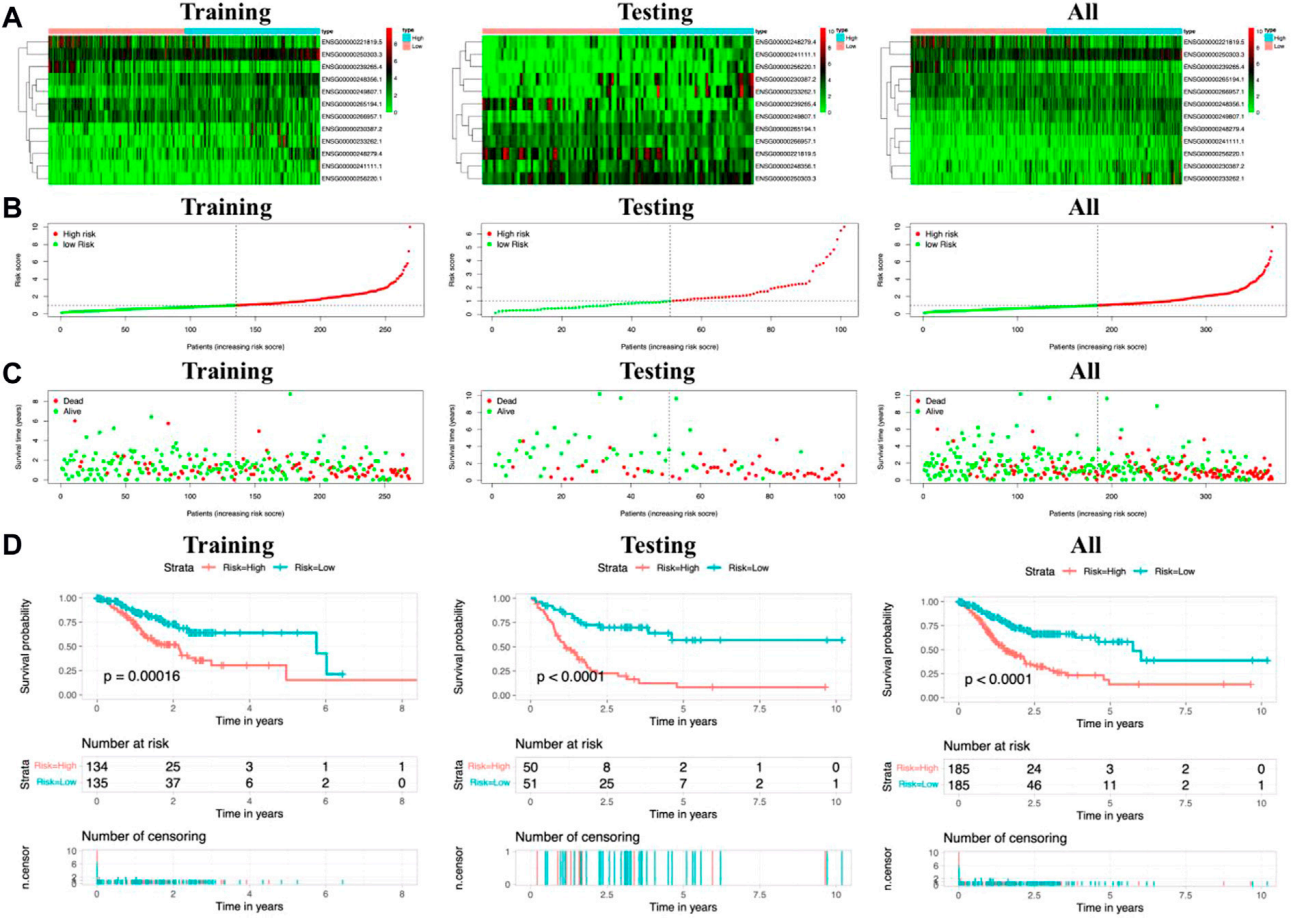
Risk scores and survival evaluation for the prognostic prediction model. **(A–C)** Prognostic signature signal heatmaps **(A)**, risk score distributions **(B)**, and overall survival (OS) status distributions **(C)** for “training,” “testing,” and “all sample” groups. **(D)** Kaplan–Meier (K-M) survival curves for patients separated into the high-risk and low-risk groups.

Based on the expression levels of 12 metal-dependent PCD-related lncRNA signatures, we calculated the risk score for each sample among the “training,” “testing,” and “all samples” groups (Figure 3A). Also, the patients were divided into high-risk (risk scores higher than the median) and low-risk (risk scores lower than the median) groups. The risk score distributions for each patient were shown in Figures 3B,C, which showed a worse survival status in the high-risk group.

**FIGURE 4:**
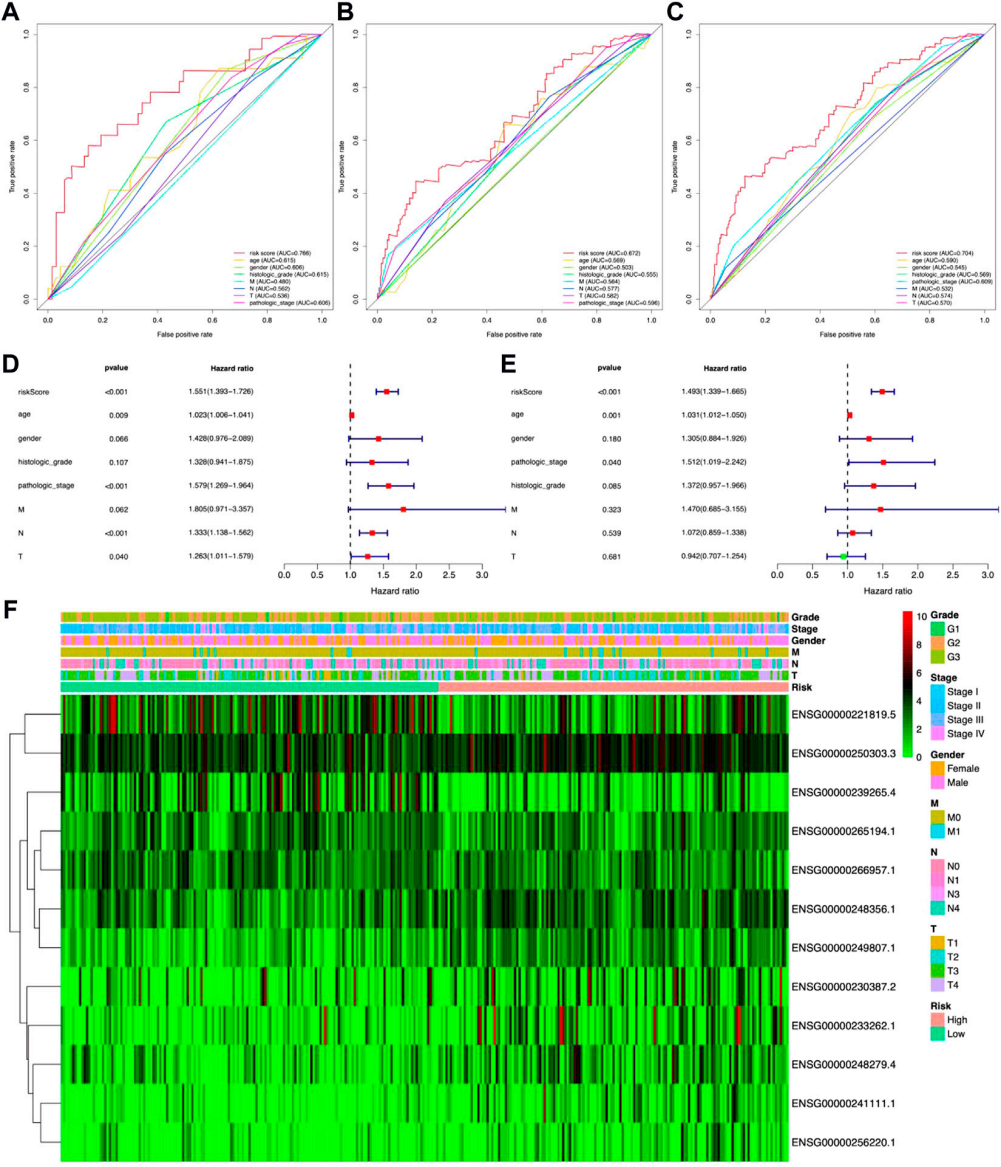
Identification ability and independence evaluation for the prognostic prediction model. **(A–C)** Receiver operating characteristic (ROC) curve analysis for the prognostic model based on 12 metal-dependent programmed cell death-related lncRNAs. **(D,E)** Forest plot for the overall survival (OS) prognostic values through univariate Cox regression analysis **(D)** and multivariate Cox regression analysis **(E)**. **(F)** Heatmap for the relationship between risk scores and clinical phenotypes of patients.

In the meantime, the prognostic effectiveness of these 12 lncRNA signatures was evaluated with K-M survival analysis. The OS rates for the high-risk group were significantly poorer than those of the low-risk group (Figure 3D, *p*-value < 0.0001). Using ROC curve analysis, the AUC value in the training group is 0.766 (Figure 4A), 0.672 in the testing group (Figure 4B), and 0.704 in all samples (Figure 4C). Then, in order to evaluate the independence of the 12 lncRNA prognostic signatures, we performed univariate and multivariate Cox regression analysis. The results of which indicated that the prognostic prediction model constructed in our work was an independent prognostic evaluation factor (HR = 1.493, 95% CI = 1.339–1.665, *p*-value < 0.001, Figures 4D,E). Additionally, the relationships between the 12 lncRNA signatures and clinicopathological features are shown with a heatmap in Figure 4F.

### Construction and evaluation of the metal-dependent programmed cell death-related lncRNA-based prognostic nomogram

**FIGURE 5:**
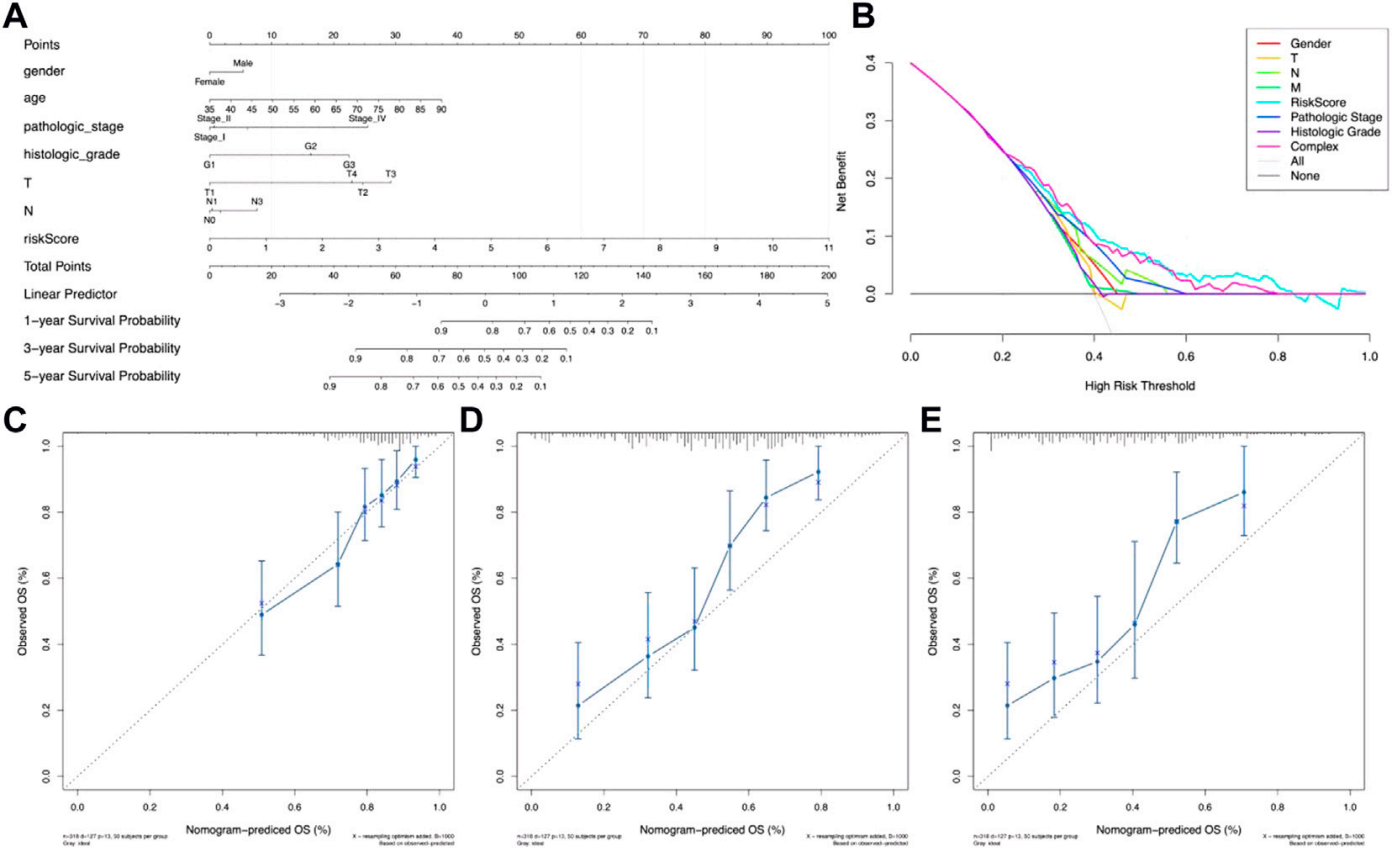
Nomogram of OS prediction of gastric cancer patients. **(A)** Nomogram for risk scores and clinical pathological factors. **(B)** Decision curve analysis (DCA) of the clinical practicality evaluation of the nomogram. **(C–E)** Calibration curve analysis of the 1-year **(C)**, 3-year **(D)**, and 5-year **(E)** survival prediction accuracy.

In order to evaluate the potential clinical practicality of the 12 metal-dependent PCD-related lncRNA signatures, a nomogram was constructed with the risk scores and the clinicopathological features for gastric cancer patients. As shown in Figure 5A, we observed that the higher the risk score calculated, the poorer the prognosis predicted. Also, the range of risk scores could cover the 1-, 3-, and 5-year overall survival rates. The DCA curve shows better clinical practicality for gastric cancer patient prognostic prediction (Figure 5B). Then, the calibration curve showed relatively good fits for the 1- (Figure 5C), 3- (Figure 5D), and 5-year OS prediction (Figure 5E).

### Tumor mutation burden, immune functions, and drug sensitivity prediction

**FIGURE 6:**
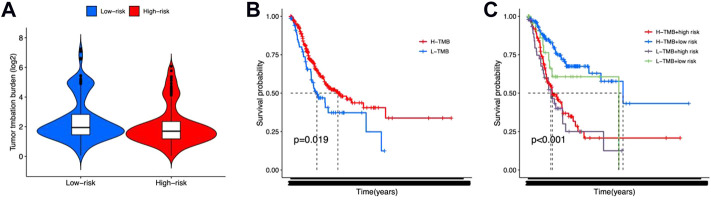
Tumor mutation burden for high-risk and low-risk groups. **(A)** Violin plot for tumor mutation burden showed no significant difference between high-risk and low-risk groups. **(B,C)** K-M survival curves shown the survival status of whether or not to combine the tumor mutation burden factors.

TMB, immune function, and drug sensitivity might also affect the outcome of gastric cancer, which may impact the prognostic prediction of the 12 lncRNA signatures. In order to evaluate the effect of TMB, we calculated the TMB scores for each gastric cancer patient and observed no significant difference between high-risk and low-risk groups (Figure 6A). The K-M survival analysis results showed that patients with high TMB scores exhibited poorer OS status (Figure 6B). Also, a combination of TMB scores may improve the OS prediction for gastric cancer (Figure 6C).

**FIGURE 7:**
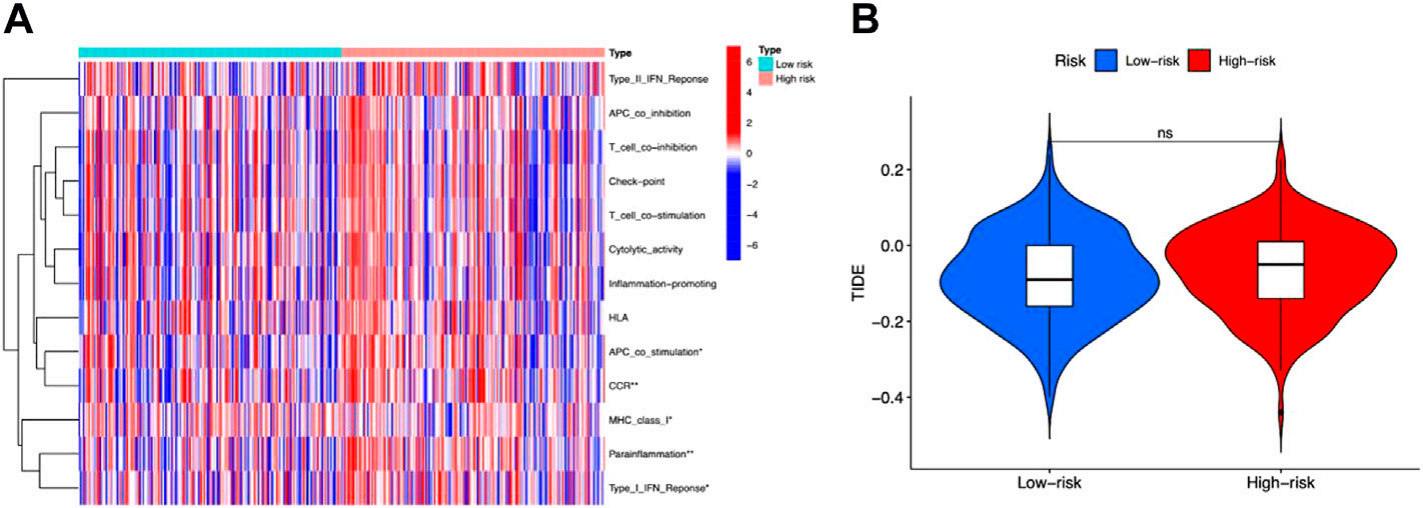
Immune functions and immunotherapy prediction. **(A)** Immune function correlation heatmap. **(B)** Immunotherapy prediction shown no significant difference between high-risk and low-risk groups.

Tumor Immune Dysfunction and Exclusion (TIDE) was then performed to evaluate immune functions and immunotherapy prediction. We observed that “APC co-stimulation,” “CCR,” “MHC class I,” “parainflammation,” and “type I IFN response” were significantly different between high-risk and low-risk groups (Figure 7A). But the TIDE scores between these two groups showed no difference (Figure 7B).

**FIGURE 8:**
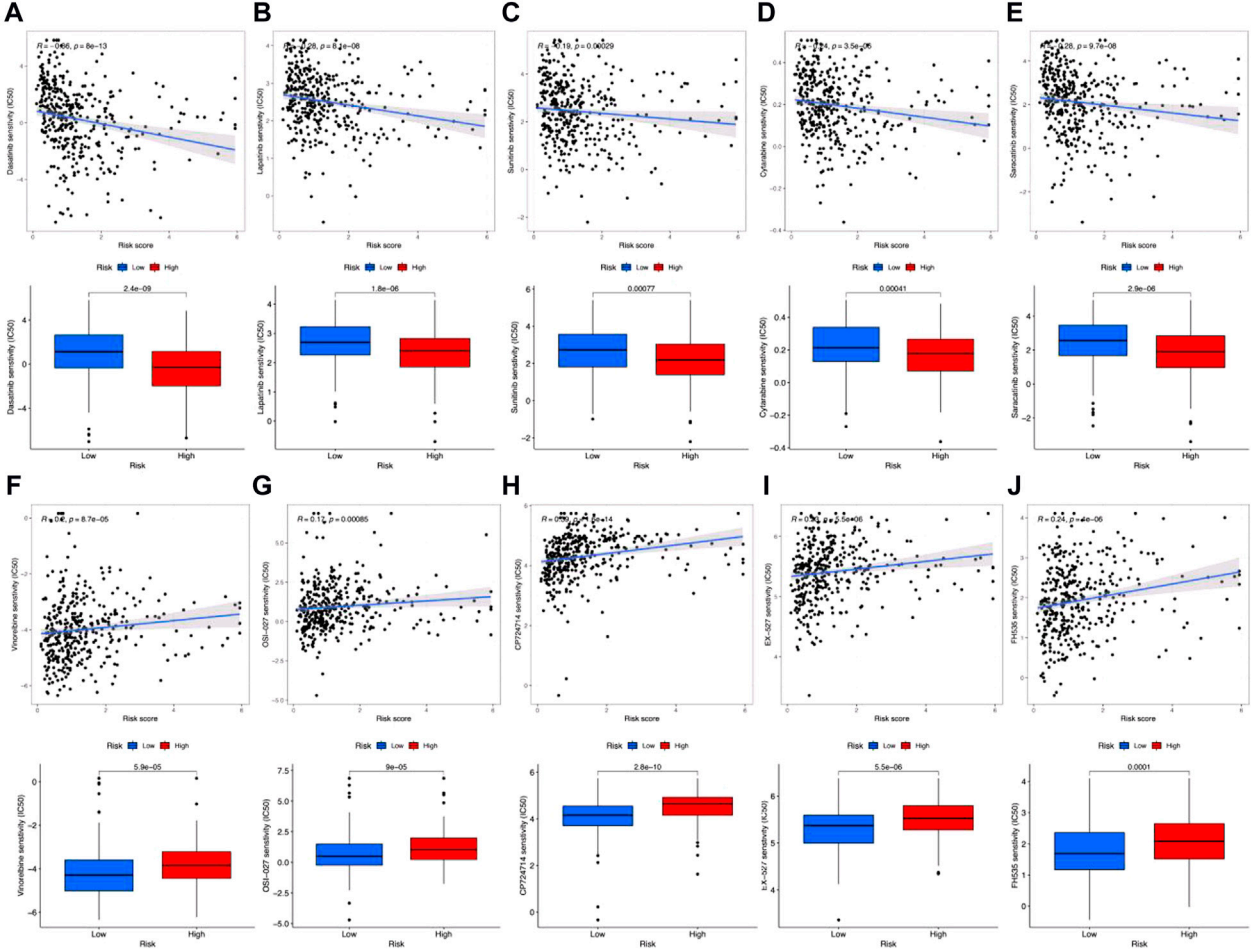
Clinically used antigastric cancer drug sensitivity prediction. **(A–J)** Correlation coefficients (up) and difference (down) between IC50 and risk scores among dasatinib **(A)**, lapatinib **(B)**, sunitinib **(C)**, cytarabine **(D)**, saracatinib **(E)**, vinorelbine **(F)**, OSI-027 **(G)**, CP724714 **(H)**, EX-527 **(I)**, and FH535 **(J)**.

Finally, we also predicted drug sensitivities for clinical antigastric cancer drugs. Results in Figure 8 show that the IC50 for dasatinib (Figure 8A), lapatinib (Figure 8B), sunitinib (Figure 8C), cytarabine (Figure 8D), and saracatinib (Figure 8E) was negatively correlated with risk scores. Also, the IC50 was remarkably lower among high-risk groups, which means that these antitumor drugs would be more sensitive for high-risk gastric cancer patients. However, IC50 for vinorelbine (Figure 8F), OSI-027 (Figure 8G), CP724714 (Figure 8H), EX-527 (Figure 8I), and FH535 (Figure 8J) was positively correlated with risk scores, which were also significantly higher in high-risk groups. In addition, the sensitivities for other antitumor drugs are shown in Supplementary Figures S3, S4. Although these results still need further clinical verification, these predictions may also be helpful for gastric cancer prognostic evaluation and individualized chemotherapy regimen design.

## Discussion

Ferroptosis and cuproptosis are newly identified metal-dependent types of programmed cell death distinct from apoptosis, autophagy, and pyroptosis (Dixon et al., 2012; Tang et al., 2022). Ferroptosis is driven by iron-triggered peroxidation while cuproptosis is a copper-dependent modality of mitochondrial cell death (Doll et al., 2017; Cobine and Brady, 2022; Li et al., 2022). Recent studies have shown that

Both ferroptosis and cuproptosis played important roles in the generation and outcome of solid tumors. Through promoting the location of DPP4 in a nonenzymatically active nucleus, TP53 could inhibit ferroptosis and promote the growth of colorectal cancer cells (Xie et al., 2017). In gastric cancer, apatinib exhibits its antitumor activity by inducing the ferroptosis process through lipid peroxidation (Zhao et al., 2021). Also, some studies showed the expression of ferroptosis- and cuproptosis-related genes and lncRNA-exhibited tumor prognostic prediction values. Pan et al. (2021) showed that the AUC value for 17 ferroptosis-related lncRNAs was 0.751 in gastric cancer. The cuproptosis-related risk score would predict the outcome of hepatocellular carcinoma (HCC) with an AUC value of 0.72 (Zhang et al., 2022b), and Zhang et al. (2022a) also constructed a cuproptosis-related lncRNA signature for HCC with a higher AUC value 0.739. But the cuproptosis-related genes and lncRNAs are still not used for gastric cancer prognostic prediction. In our study, both ferroptosis- and cuproptosis-related genes and lncRNAs were involved in the construction of the gastric cancer prognostic prediction model. We have identified 12 lncRNA prognostic signatures for gastric cancer outcome status evaluation. ROC curve analysis shows the AUC value for this model reached 0.766 in our study, which is higher than other published signatures. The model by Pan et al. (2021) model showed a high AUC value of 0.751. Chen et al. (2021) obtained the 0.736 AUC value using autophagy-related genes. In addition to this, the AUC of the model by Jiang et al. (2021) was 0.654 at 1 year, 0.657 at 3 years, and 0.733 at 5 years. In addition, the AUC values for much more related works were no more than 0.7 (Hu et al., 2019; Yu et al., 2019; Qi et al., 2020). Therefore, these results indicate that the 12 metal-dependent programmed cell death lncRNA signatures constructed in our study improved the prognostic evaluation effectiveness for gastric cancer.

Antitumor drug sensitivities would also determine the outcome of gastric cancer. For instance, as reported, the small molecular targeted drug CP724714 may decrease the IC50 and drug resistance index of gastric cancer to cisplatin (Huang et al., 2016). Dasatinib is an efficient gastric cancer inhibitor, which targets SRC family kinases (Montenegro et al., 2020). EX-527 is a specific inhibitor to SIRT1, which would reintroduce the chemotherapeutic sensitivity (Zhu et al., 2012). FH535 combined with taxol can enhance the invasion inhibitory effect on gastric cancer (Liu et al., 2018). Lapatinib is a kind of tyrosine kinase inhibitor that significantly decreases cell viability and migration of gastric cancer cells, which can also induce cell apoptosis suffering G0/G1 arrest (Yi et al., 2021). R_8_-modified vinorelbine combined with schisandrin B liposomes can significantly inhibit gastric cancer metastasis by downregulating VEGF, VE-Cad, HIF-1a, PI3K, MMP-2, and FAK (Li et al., 2021). Sunitinib is a multitargeted tyrosine kinase inhibitor, which can enhance the cytotoxicity of vincristine, adriamycin, and cisplatin on multidrug resistant gastric cancer cells (Zhang and Wang, 2013; Hojo et al., 2022). Saracatinib is an Src inhibitor that suppresses gastric cancer invasion and migration, which can also enhance the antitumor effects of lapatinib (Bertotti et al., 2010; Nam et al., 2013; Yamaguchi et al., 2014). Saracatinib also has a synergistic effect with trastuzumab on antigastric cancer (Han et al., 2014). Moreover, OSI-027 can enhance oxaliplatin-induced cell apoptosis and inhibit multidrug resistance to gastric cancer (Xu et al., 2021). In our study, we predicted IC50 for dasatinib, lapatinib, sunitinib, cytarabine, and saracatinib were negatively correlated with risk scores. These results demonstrated that these antitumor drugs would be more sensitive in the high-risk group, which may be more suitable for clinical usage toward patients in the high-risk group. Meanwhile, IC50 for vinorelbine, OSI-027, CP724714, EX-527, and FH535 was positively correlated with risk scores, which demonstrated these drugs may be unsuitable.

Some studies have shown that Chinese patent medicines and plant extracts can also induce apoptosis through ferroptosis and cuproptosis. Liu et al. (2022) found that disulfiram (DSF)/Cu elevated the generation of reactive oxygen species (ROS), and apoptosis was induced in a ROS-dependent manner. Song et al. (2022) showed that Yiqi Huayu decoction can induce ferroptosis in GC by affecting the JAK2-STAT3 pathway and the expression of ACSL4. Guan et al. (2020) found that tanshinone IIA could suppress the proliferation of gastric cancer *via* inducing p53 upregulation-mediated ferroptosis. Other studies have found that salidroside can inhibit the growth of gastric cancer and induce apoptosis through the PI3K/Akt/mTOR pathway (Rong et al., 2020). Therefore, traditional Chinese medicine and plant extracts also play an important role in the treatment of gastric cancer. Some studies have also shown that TCM extracts can fight cancer by promoting programmed cell apoptosis. Tanshinone IIA (Tan-IIA) was extracted from Danshen (Salviae miltiorrhizae Radix). Su (2018) found that Tan-IIA inhibited human gastric cancer AGS cells; one of the molecular mechanisms may be through decreasing the protein expression of VEGFR and HER2, then blocking the Ras/Raf/MEK/ERK pathway to induce the activation of PARP and caspase-3 to induce apoptosis. ’It is worth mentioning that Ni et al. (2022) and Guan et al. (2020) pointed out that Tanshinone IIA inhibited the stemness of gastric cancer cells partly by inducing ferroptosis. Chen et al. (2017) showed that the tuber of Amorphophallus konjac could increase cell apoptosis and induce cell cycle arrest. TuAKe could also promote autophagy. Moreover, toosendanin (TSN) is a triterpenoid derived from the bark of Melia toosendanin Sieb. Zhou et al. (2018) found that TSN suppressed cell viability, inhibited cell proliferation by causing G1/S arrest, and induced caspase-dependent apoptosis in AGS and HGC-27 cells. In addition, macrocalyxin C is a Chinese herb-derived diterpenoid compound that has been postulated to possess anticancer characteristics. Dang et al. (2020) showed that macrocalyxin C may halt the proliferation of gastric malignancies through alteration of cell invasion, apoptosis, progression through the cell cycle, and cell growth.

All in all, we screened differentially expressed metal-dependent programmed cell death-related lncRNAs and constructed a 12-lncRNA prognostic prediction model for gastric cancer, the prognostic power of which was better than that of other models. We observed that tumor mutation burden and immunotherapy effect have no difference between the high-risk and low-risk groups. Also, antitumor drug sensitivity analysis results may also be helpful for individualized chemotherapy regimen design.

## Data Availability

The datasets presented in this study can be found in online repositories. The names of the repository/repositories and accession number(s) can be found in the article/Supplementary Material.

## Supplementary material

Supplementary Figure S1 Flowchart for this work.

Supplementary Figure S2 Forest plot for the prognostic values of the metal-dependent programmed cell death-related lncRNA signatures through univariate Cox regression analysis.

Supplementary Figure S3 Correlation coefficients between IC50 and risk scores for other antitumor drugs.

Supplementary Figure S4 Difference of IC50 between high-risk and low-risk groups for other antitumor drugs.
