# Supplementary figures and images for: Metal-dependent programmed cell death-related lncRNA prognostic signatures and natural drug sensitivity prediction for gastric cancer
Source: Front Pharmacol. 2022 Oct 21;13:1039499. doi: 10.3389/fphar.2022.1039499 (PMC9634547; doi:10.3389/fphar.2022.1039499)

**Figure S1**

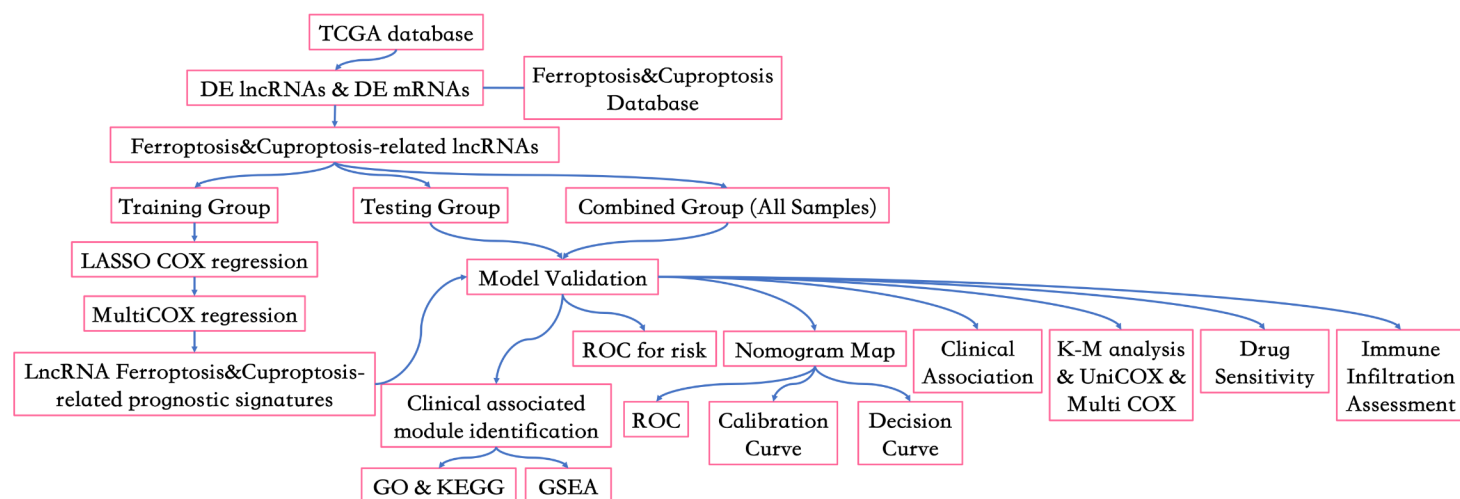

Figure S2

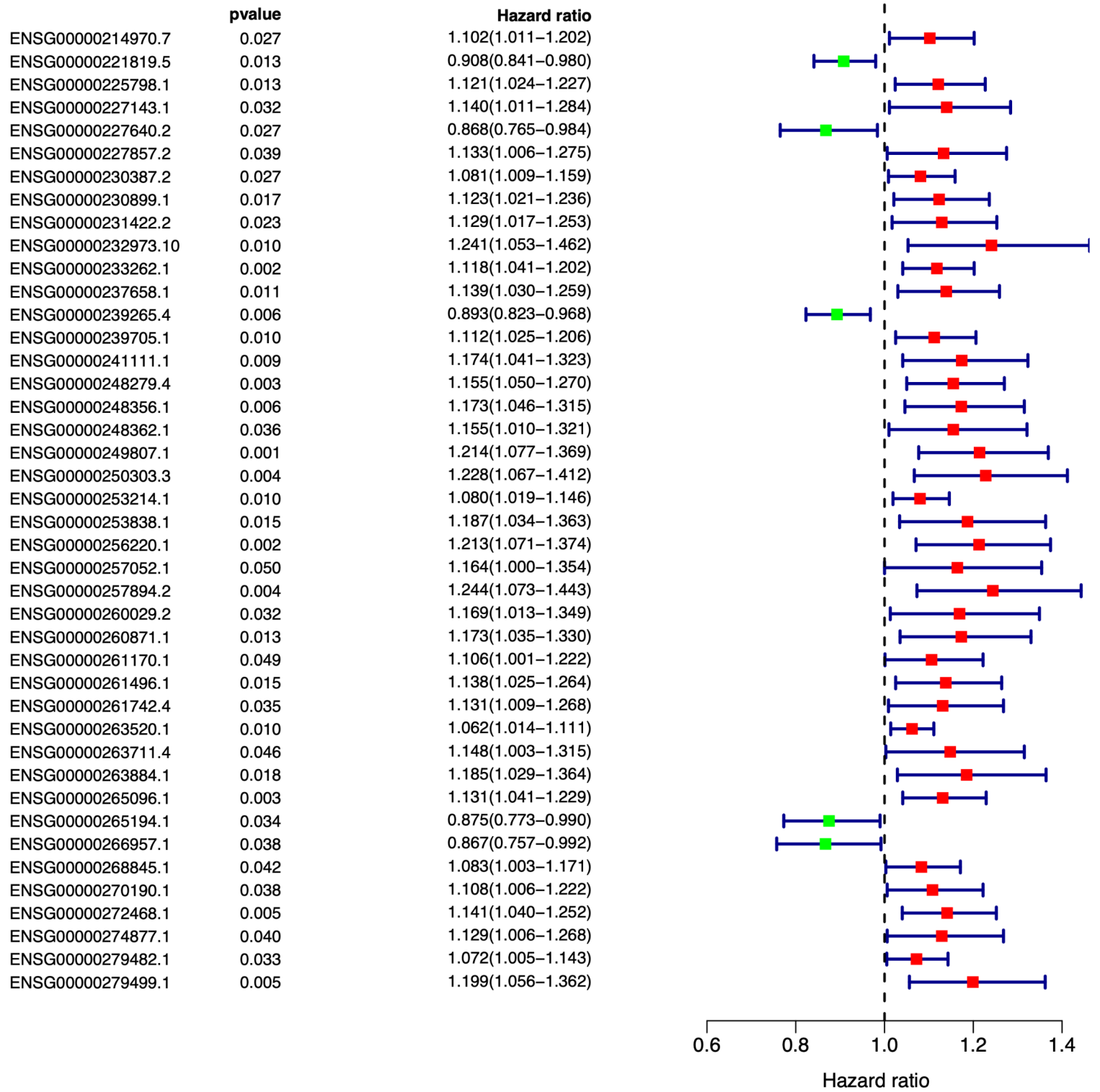

Figure S3

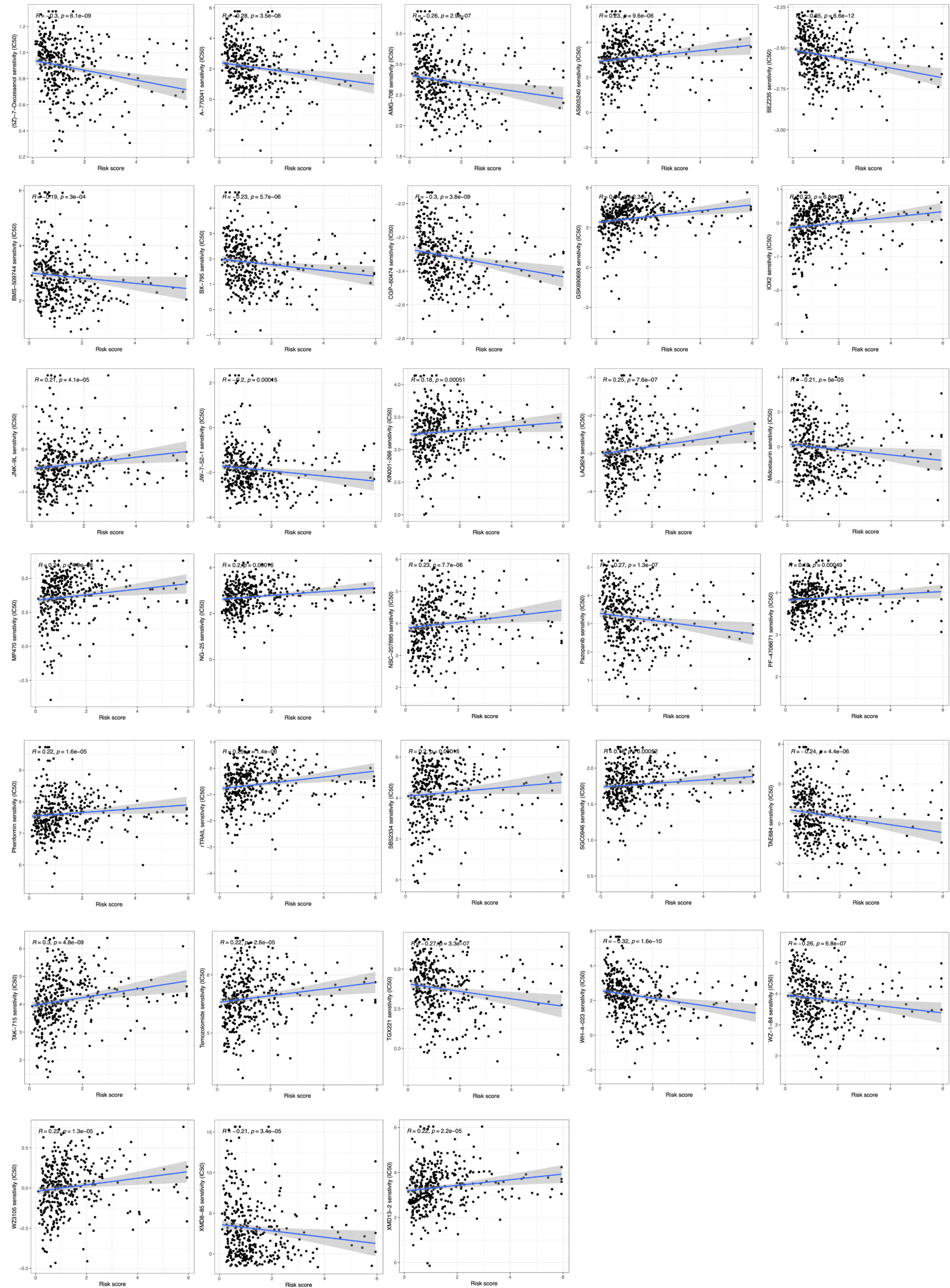

Figure S4

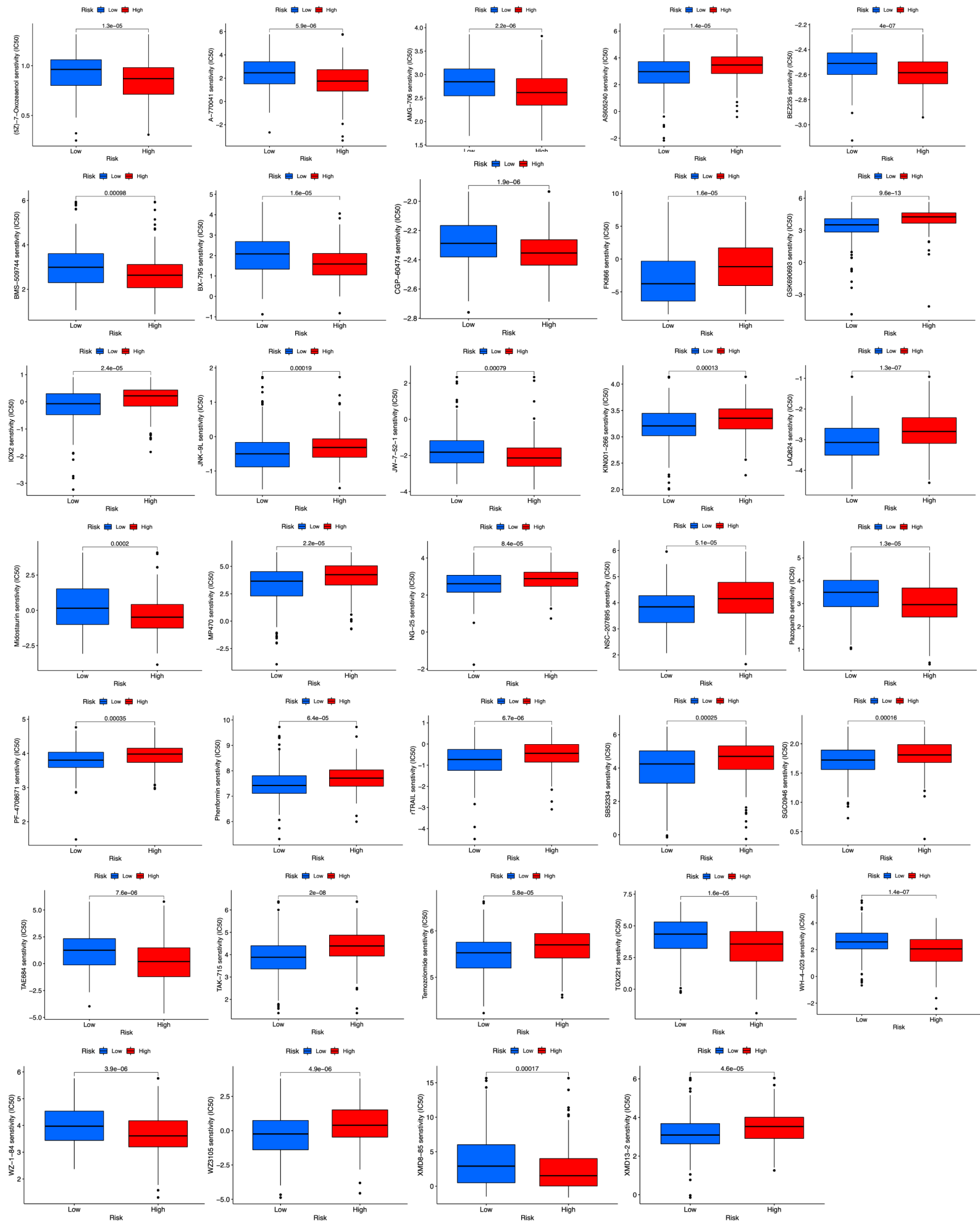

Supplement: Supplementary file 1 [file DataSheet1.PDF]
